# Regional Differences in COVID-19 Mortality Rates in the Kingdom of Saudi Arabia: A Simulation of the New Model of Care

**DOI:** 10.7759/cureus.20797

**Published:** 2021-12-29

**Authors:** Abdullah A Alharbi, Ahmad Y Alqassim, Mohammed A Muaddi, Saleh S Alghamdi

**Affiliations:** 1 Family and Community Medicine Department, Faculty of Medicine, Jazan University, Jazan, SAU; 2 Clinical Audit General Directorate, Ministry of Health, Riyadh, SAU

**Keywords:** saudi arabia, healthcare policy, regional differences, environmental health, healthcare system, covid-19, mortality

## Abstract

Background

This study aimed to assess regional COVID-19 mortality rates and compare the five proposed business units (BUs).

Methods

A cross-sectional study was conducted in the Ministry of Health (MOH) hospitals in the Kingdom of Saudi Arabia (KSA). We included 1743 adults (≥ 18 years of age) with COVID-19 admitted to any of 30 MOH hospitals.

Results

The inpatients had confirmed mild to severe COVID-19 between March and mid-July 2020. The central BU (Riyadh) was used as the reference. MOH electronic health record data were reviewed and utilized, including variables reflecting hospital course (mortality and discharge status). The primary outcome was COVID-19-related inpatient death. Covariates included patient demographics, pre-existing chronic diseases, and COVID-19-related complications. The data were analysed using univariate and multivariate logistic regression. KSA inpatient mortality was 30%. Univariate and multivariate logistic regression analysis suggested that COVID-19-related mortality was significantly higher in the northern and western BUs and significantly lower in the southern and eastern BUs than in the central BU. On controlling for other variables, adjusted odds ratios (AORs) for essential COVID-19 mortality predictors during admission, using the central BU as a reference, were as 9.90 [95% CI, 4.53-21.61] and 1.55 [95% CI, 1.04-2.13] times higher in the northern and western BUs, respectively, and 0.60 [95% CI, 0.36-0.99] and 0.23 [95% CI, 0.14-0.038] times lower in the southern and eastern BUs, respectively.

Conclusion

The five BUs differed in COVID-19 mortality rates after adjusting for patient and disease characteristics, with the differences consistent with those in the regions comprising the BUs. These outcome differences apparently relate to differences in healthcare resources and quality.

## Introduction

On 5 January 2021, the global mortality rates of the 2019 coronavirus disease (COVID-19) pandemic, caused by SARS-CoV-2, were approximately 2.2%, based on 1,864.933 deaths among 86,298,213 cases [1-3]. In the Kingdom of Saudi Arabia (KSA), as of 6 January 2021, the national average mortality rate for all cases, both inpatient and outpatient, was 1.7%, based on 6,272 deaths among 363,377 confirmed cases [4]. The COVID-19 death rate has been increasing in the KSA from 0.83% in June 2020 to 1.24% in August 2020, and to 1.7% in January of 2021. Although, variations in COVID-19 mortality rates between different KSA regions have been reported. mortality rates in the KSA remain among the lowest globally, for example, compared to the UK’s 15% [5].

Several international studies have identified risk factors associated with higher mortality from confirmed COVID-19 infection, including increasing age and pre-existing chronic conditions, such as Type 2 diabetes mellitus (DM2), cardiovascular disease, and obesity. However, a wide variation in global mortality rates has been reported [6, 7]. Studies including all fifty states in the United States have shown higher mortality rates in states with a higher average age [8]. Studies from China, Italy, and Mexico have demonstrated that sex, age, smoking, and comorbidities, such as type 2 diabetes mellitus (DM2), hypertension (HTN), kidney disease, and cardiac disease, are related to increased risk of mortality from COVID-19 [9-12]. Geriatric patients may have increased mortality risks as their pre-existing conditions increase and have higher requirements for interventions such as oxygen, ICU, mechanical ventilation, and longer hospital stays [13-16]. A study of ICU admissions for COVID-19 inpatients in the KSA revealed age, sex, and comorbidities as risk factors similar to international studies [17]. Based on the aforementioned study and a review of international studies it is reasonable to expect that the elderly in Saudi Arabia would also be at higher risk for mortality [18-21]. Other studies have demonstrated a relationship between the quality and resources of healthcare systems and mortality rates; for example, experiences from previous pandemics like H1N1 influenza in 2009 showed that, whereas age is a factor, other factors, such as disparities in income, access to healthcare resources, education levels, and overall population health, impacted mortality rates during that pandemic [22, 23]. A study that examined mortality risk factors in COVID-19 patients both in and out of hospitals in Germany, Spain, Italy, and the USA concluded that the primary risk factors for mortality were the lack of intensive care unit (ICU) beds and ventilation devices [24]. Studies of long-term care facilities concluded that the most substantial risk factors for mortality were quality-of-care factors, such as overcrowding, staffing levels, availability and use of personal protective equipment for staff, nosocomial transmission of COVID-19 from staff to residents due to high care needs, sex, functionality level, immune and inflammatory responses, and the course of the disease, whereas comorbidities were not significant risk factors [25-27]. Regional disparities in general healthcare quality in Saudi Arabia have been previously reported, including resource allocation, staffing, technology, and care standards [28].

The present public healthcare system is no longer sustainable due to increasing demand for services, population aging, and an economic downturn [29]. Therefore, the KSA is launching a new model of care (MOC) as part of the Vision 2030 sustainable development initiative that will privatize healthcare administration as the optimum means to improving the healthcare system's efficiency, quality, and value [30]. Although privatization implies a transfer of ownership to a private company from a government, the new MOC in the KSA will be a hybrid model, whose transformation will be directed by the Vision Realization Office of the Ministry of Health (MOH) [30, 31]. The novel healthcare system will have a division of responsibilities in which the five business units (BUs) to be formed through the consolidation of the present 13 regions will be administered by five private insurance companies responsible for the actual healthcare of each BU under a government-owned holding company. The healthcare system's financial administration will continue to be under the oversight of the MOH, and a national insurance center will have the task of ensuring that all Saudis have insurance coverage [30]. This study aimed to assess COVID-19-related mortality rates within regions and compare the rates between the five proposed BUs to be implemented under Vision 2030. The ultimate intention was to utilize COVID-19 mortality rates as an indicator of healthcare quality to inform national healthcare reform efforts. We hypothesized that variations in mortality rates among the new BU's would reflect variations between the regions of which each BU will be composed.

## Materials and methods

Study design and population

We conducted a cross-sectional study using national MOH electronic-health-record data on COVID-19-related deaths. Our study population comprised 1743 adults (men and women ≥ 18 years of age) with COVID-19 admitted to any of the 30 MOH hospitals in the 13 administrative regions of KSA. We investigated the mortality rates of inpatients with confirmed moderate-to-critical COVID-19 as defined by the MOH between March 2020 and mid-July 2020. A specific protocol was used to classify patients with COVID-19 in the KSA. The MOH provided classification protocols for the severity of COVID-19 cases based on the Centers for Disease Control (CDC) as follows: critical - respiratory failure, severe - low oxygen saturation, moderate - adequate oxygen saturation in the presence of lower respiratory disease, and mild - asymptomatic [32].

Healthcare settings in KSA

The KSA is divided into 13 administrative regions that currently serve a population of 34,218,169 with a blend of public and private sectors. During the COVID-19 pandemic, all COVID-19 patients were treated free of charge at Public Health Healthcare Centers (PHCCs), which include both public and private hospitals. The 30 MOH hospitals were distributed throughout the present 13 health administrative regions of the KSA. We aggregated the data into the five proposed BUs that will be constituted under Vision 2030 to simulate mortality rates and quality-of-care projections under the new model of care (MOC).

Data source and measures 

MOH electronic medical record (EMR) data on COVID-19-related mortality was reviewed and utilized. Data obtained for this study included the hospital clinical course (mortality and discharge status). The primary outcome was COVID-19-related inpatient death and was coded as a binary variable for analysis purposes as follows: 0 = did not die in a hospital, and 1 = did die in a hospital. Covariates included the three categories of patient demographics, pre-existing chronic diseases, and COVID-19-related complications. Demographics included age, sex, and nationality. Pre-existing diseases included DM2, hypertension (HTN), cardiac diseases, obesity, acute respiratory distress syndrome (ARDS), and others. Complications from COVID-19 included ARDS, bacterial pneumonia, sepsis, multiorgan failure, and others. The BU or main region in which a patient was admitted was the main independent categorical variable of interest. BUs were coded as follows: 0 = Central (Riyadh and AL Qassim regions), 1 = Eastern (Sharqiyah region only), 2 = Western (Makkah, Medina, and Al Baha regions), 3 = northern (Al Jawf, Hail, Northern Frontier, and Tabuk regions), and 4 = Southern (Asir, Jazan, and Najran regions).

Statistical analysis 

Data were analyzed using several statistical techniques as appropriate. The means and standard deviations of continuous variables were assessed using one-way ANOVA and independent samples t-test. The Chi-square test was used to compare the proportions of categorical variables. The primary outcome, COVID-19 mortality, was analyzed using multivariate and univariate logistic regression because of its dichotomous nature. Univariate analysis was conducted for BUs only because they were our primary focus. To understand the reasons behind any differences in mortality rate, we used multivariate analysis, where the covariates included sex, nationality, age group, history of chronic diseases, complications, and length of stay. We selected the central BU as a reference point because it has the most available resources, such that any resource disparity would be more discernible. STATA-14 (Stata Corp, College Station, TX, USA) statistical software was used for data analysis, and all tests were two-sided with statistical significance set at a p-value < 0.05.

Ethical consideration

This study was conducted as per the National Committee of Bioethics (NCBE) guidelines, Saudi Arabia. Further, the Central Institutional Review Board provided ethical approval of the Saudi MOH study (reference number: 20-163E). The study relied on secondary data collected from the MOH, and the privacy and confidentiality of the data were strictly maintained. All data were fully anonymized before accessing them and the national NCBE waived the requirement for informed consent since data were retrieved from medical records.

## Results

**Figure 1:**
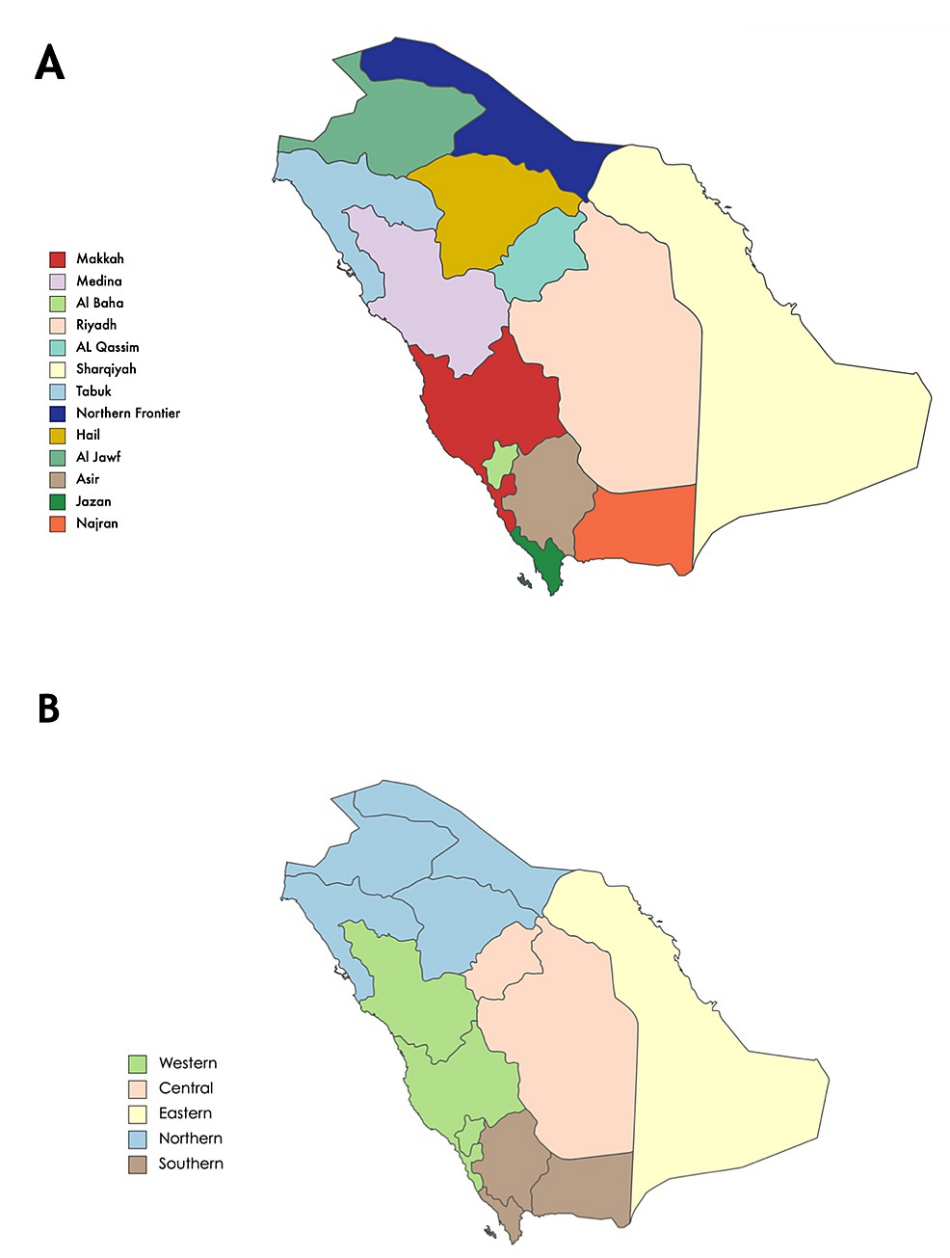
The administrative geographical distribution in the Saudi model of care. A) The current administrative regions in the current Saudi model of care. B) The five proposed business units in the new Saudi model of care

**Figure 2:**
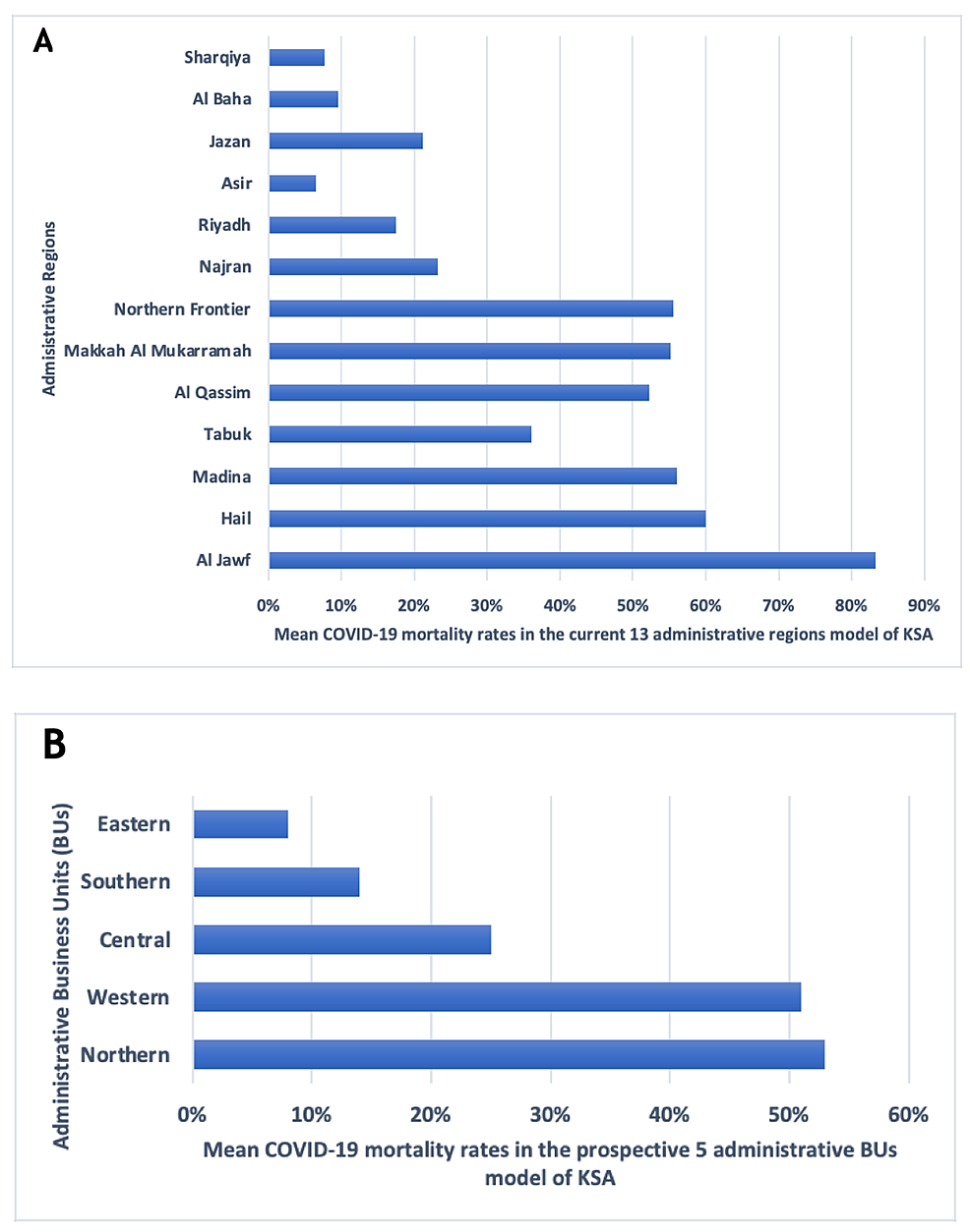
The COVID-19 mortality rates in the Saudi model of care. A) The COVID-19 mortality rates the current Saudi model of care. B)  The COVID-19 mortality rates in the new Saudi model of care

Table 1, Figure 1 and Figure 2 present the demographic characteristics of the 1743 participants according to the different BUs and geographical regions in the five KSA BUs. Men represented 71% of the total sample and ranged from 60% to 85% among the BUs. Non-Saudi participants constituted 52% of the total sample, although the distribution varied by BU. Participant nationality was predominantly non-Saudi in the central and western BUs and Saudi in the northern, southern, and eastern BUs. Within BUs, there were significant demographic differences between regions. Men predominated in all BUs but by differing ratios. Al Baha in the western BU was the only region with equal male and female participants, and the most significant gaps between male and female participants occurred in the central (15-85%) and northern (19-81%) BUs. Most participants in all BUs were in the 40-59-year age group, except for those in Al Baha in the western BU who predominantly occupied the 19-39-year age group and those in Hail and Northern Frontier who were mostly over 60 years of age. Compared to other BUs, the western and northern BUs had the most significant proportions of participants over the age of 60, that is, 32% and 38%, respectively.

**Table 1:** Demographic profiles of COVID-19 inpatients in the regions of the five BUs of the KSA *BUs are the main proposed new sectors; regions are the 13 regions of the KSA. BU: business unit; KSA: Kingdom of Saudi Arabia

Business Unit*	Regions	Gender		Age Groups (years)			Nationality		All patients N (%)
		Male N (%)	Female N (%)	19–39 N (%)	40–59 N (%)	60+ N (%)	Non-Saudi N (%)	Saudi N (%)	
Total	All regions	1239 (71)	538 (29)	495 (29)	806 (46)	442 (25)	914 (52)	829 (48)	1743 (100)
Central	Al Qassim	56 (84)	11 (16)	13 (19)	31 (47)	23 (34)	44 (66)	23 (34)	307 (18)
	Riyadh	204 (85)	36 (15)	67 (28)	137 (57)	36 (15)	194 (81)	46 (19)	
	All Central	260 (85)	47 (15)	80 (26)	168 (55)	59 (19)	238 (78)	69 (22)	
Western	Al Baha	27 (52)	25 (48)	35 (67)	10 (19)	7 (13)	5 (10)	47 (90)	655 (37)
	Makkah Al Mukarramah	403(72)	159 (28)	93 (17)	275 (49)	194 (34)	374 (67)	188 (33)	
	Madina	35 (85)	6 (15)	7 (17)	22 (54)	12 (29)	32 (78)	9 (22)	
	All Western	465 (71)	190 (29)	135 (21)	307 (47)	213 (32)	411 (63)	244 (37)	
Northern	Al Jawf	4 (100)	0 (00)	1 (25)	3 (75)	0 (00)	3 (75)	1 (25)	91 (5)
	Hail	26 (74)	9 (26)	8 (23)	13 (37)	14 (40)	10 (29)	25 (71)	
	Northern Frontier	24 (89)	3 (11)	1 (4)	12 (44)	14 (52)	13 (48)	14 (52)	
	Tabuk	20 (80)	5 (20)	7 (28)	11 (44)	7 (28)	19 (76)	6 (24)	
	All northern	74 (81)	17 (19)	17 (19)	39 (43)	35 (38)	45 (49)	46 (51)	
Southern	Asir	68 (73)	25 (27)	23 (25)	54 (58)	16 (17)	39 (42)	54 (58)	188 (11)
	Jazan	39 (75)	13 (25)	17 (33)	27 (52)	8 (15)	20 (38)	32 (62)	
	Najran	31 (72)	12 (28)	7 (16)	20 (46)	16 (37)	20 (47)	23 (53)	
	All southern	138 (73)	50 (27)	47 (25)	101 (54)	40 (21)	79 (42)	109 (58)	
Eastern	Sharqiya	302 (60)	200 (40)	216 (43)	191 (38)	95 (19)	141 (28)	361 (72)	502 (29)

Table 2 presents the comorbidities, complications, and outcomes of COVID-19 inpatients according to the main five BUs of the KSA. The mean participant age was 50 years. Generally, there was a significant variation in the history of chronic diseases across BUs. All combined chronic diseases and mean age were statistically significantly lower in the eastern BU than in the central and all other BUs (p < 0.001 for all). Compared to those in the central BU, mean age and the overall rate of chronic diseases were significantly higher in the western and northern BUs (p < 0.001 for all). Compared to that in the central BU, the presence of ARDS on admission was significantly higher in the northern BU and significantly lower in all other BUs. The clinical outcomes of COVID-19 inpatients by BU revealed significant variation. The overall COVID-19-related mortality rate for all inpatients was 30%, with significant differences between BUs. Compared to the central BU, the western and northern BUs had statistically significantly higher mortality rates, that is, more than double. In comparison, the eastern and southern BUs had significantly lower rates at one-third and approximately half of those of the central BU, respectively. The total sample revealed that the most frequent COVID-19-related clinical complications were bacterial pneumonia (39%), ARDS (31%), sepsis (21%), acute kidney injury (14%), and multiorgan failure (10%), with all others occurring in less than 10% of patients. There was a statistically significant variation in the frequency of complications between the BUs. Compared to the central BU, the western BU experienced significantly higher rates of all complications, except bacterial pneumonia and arrhythmia, whereas the northern BU had a significantly higher rate of hepatotoxicity only. The rate of complications was lower in the eastern BU than in all other BUs, including the central BU. Compared to the central BU, the western and northern BUs had significantly higher ICU-admission rates, whereas the eastern and Southern BUs had significantly lower rates.

**Table 2:** Clinical course, complications, and outcomes of COVID-19 inpatients according to the five BUs of the KSA SD=standard deviation; ICU=intensive-care unit; ARDS=acute respiratory distress syndrome; GIT=gastrointestinal tract; BU=business unit; KSA=Kingdom of Saudi Arabia *Chi-square test; #p-value based on the one-way analysis of variance (ANOVA) test.

Characteristics	Total N (%)	Central N (%)	Western N (%)	Eastern N (%)	Northern N (%)	Southern N (%)	p-value*
Comorbidities							
Diabetes							<0.001
No	1009 (58)	149 (49)	345 (52)	370 (74)	42 (46)	103 (55)	
Yes	734 (42)	158 (51)	310 (47)	132 (26)	49 (54)	85 (45)	
Hypertension							<0.001
NO	1177 (68)	216 (70)	400 (61)	374 (75)	53 (58)	134 (71)	
Yes	566 (32)	91 (30)	255 (39)	128 (25)	38 (42)	54 (29)	
Obesity							<0.001
NO	1491(86)	256 (83)	503 (77)	484 (96)	84 (92)	164 (87)	
Yes	252 (14)	51 (17)	152 (23)	18 (4)	7 (8)	24 (13)	
Cardiac Diseases							<0.001
No	1513(87)	27 (90)	526 (80)	463 (92)	78 (86%)	169 (90)	
Yes	230 (13)	30 (10)	129 (20)	39 (8)	13 (14)	19 (10)	
Immunocompromised							<0.001
No	1673 (96)	298 (97)	617 (94)	494 (98)	80 (88)	184 (98)	
Yes	70 (4)	9 (3)	38 (6)	8 (2)	11 (12)	4 (2)	
History of cancer							<0.001
No	1700 (97.5)	299 (97)	629 (96)	495 (99)	89 (98)	188 (100)	
Yes	43 (2.5)	8 (3)	26 (4)	7 (1)	2 (2)	0 (0)	
ARDS at admission							<0.001
NO	1531 (88)	249 (81)	547 (84)	491 (98)	72 (79)	172 (91)	
Yes	212 (12)	58 (19)	108 (16)	11 (2)	19 (21)	16 (9)	
ICU Admission							<0.001
NO	881 (50.50)	127 (41)	191 (29)	440 (88)	11 (12)	112 (60)	
Yes	862 (49.50)	180 (59)	464 (71)	62 (12)	80 (88)	76 (40)	
Complications							
Sepsis							<0.001
No	1376 (79)	237 (77)	455 (69)	54 (90)	71 (78)	163 (87)	
Yes	367 (21)	70 (23)	2009 (31)	52 (10)	20 (22)	25 (13)	
Bacterial Pneumonia							<0.001
No	1063 (61)	152 (50)	343 (52)	423 (84)	49 (54)	96 (51)	
Yes	680 (39)	155 (50)	312 (48)	79 (16)	42 (46)	92 (49)	
ARDS							<0.001
No	1209 (69)	205 (67)	332 (51)	464 (92)	61 (67)	147 (78)	
Yes	534 (31)	102 (33)	323 (49)	38 (8)	30 (33)	41 (22)	
Arrythmia							<0.001
No	1612 (92)	269 (88)	581 (89)	500 (99.5)	80 (88)	182 (97)	
Yes	131 (8)	38 (12)	74 (11)	2 (0.5)	11 (12)	6 (3)	
Acute kidney injury							<0.001
No	1495 (86)	269 (88)	478 (73)	497 (99)	85 (93)	166 (88)	
Yes	248 (14)	38 (12)	177 (27)	5 (1)	6 (7)	22 (12)	
Hepatotoxicity							<0.001
No	1702 (98)	303 (99)	629 (96)	501 (100)	87 (96)	182 (97)	
Yes	41 (2)	4 (1)	26 (4)	0 (0)	4 (4)	6 (3)	
GIT perforation							<0.001
No	1709 (98)	306 (100)	623 (95)	501 (100)	91 (100)	188 (100)	
Yes	34 (2)	0 (0)	32 (5)	0 (0)	0 (0)	0 (0)	
Multi-organ failure							<0.001
No	1565 (90)	283 (92)	525 (80)	500 (99.50)	83 (91)	174 (93)	
Yes	179 (10)	24 (8)	130 (20)	2 (.50)	8 (9)	14 (7)	
Death in Hospital							<0.001
No	1218 (70)	231 (75)	320 (49)	463 (92)	43 (47)	161 (86)	
Yes	525 (30)	76 (25)	335 (51)	39 (8)	48 (53)	27 (14)	
Age, mean (SD) years	50 (0.38)	49.05 (13.26)	53 (15.97)	45 (16.28)	55 (16.08)	49 (14.44)	<0.001#
Duration of stay in days, mean (SD)	12 (0.2)	13.22 (8.1)	13.32 (8.2)	9.33 (7.8)	12.56 (7.7)	10.93 (8.4)	<0.001#

Table 3 and Figure 2 present a comparison of characteristics among inpatients who died of COVID-19. There were statistically significant differences regarding demographic characteristics; regional rates of deceased inpatients were 53%, 51%, 25%, 14%, and 8% in the northern, western, central, southern, and eastern BUs, respectively. Compared to women, the mortality rate for men was 40% higher, while compared to Saudis, the mortality rate of non-Saudis was 70% higher.

**Table 3:** Demographic and clinical variables associated with mortality rates among COVID-19 inpatients in the KSA. ICU=intensive-care unit; ARDS=acute respiratory distress syndrome; GIT=gastrointestinal tract; KSA=Kingdom of Saudi Arabia. *Chi-square test; #p-value based on two independent t-tests.

Characteristics	Did Not Die during admission N (%)	Died during admission N (%)	p-value*
Business Unit			<0.001
Central	231 (75)	76 (25)	
Western	320 (49)	335 (51)	
Eastern	463 (92)	39 (8)	
Northern	43 (47)	48 (53)	
Southern	161 (86)	27 (14)	
Gender			<0.001
Male	835 (67)	404 (33	
Female	383 (76)	121 (24)	
Nationality			<0.001
Non-Saudi	571 (62)	343 (38)	
Saudi	647 (78)	182 (22)	
ICU admission			<0.001
No	865 (98)	16 (2)	
Yes	353 (41)	509 (59)	
History of chronic diseases			
Diabetes			<0.001
No	778 (77)	231 (23)	
Yes	440 (60)	294 (40)	
Hypertension			<0.001
No	882 (75)	295 (25)	
Yes	336 (59)	230 (41)	
Obesity			<0.001
No	1093 (73)	398 (27)	
Yes	125 (50)	127 (50)	
Cardiac Diseases			<0.001
No	1106 (73)	407 (27)	
Yes	112 (49)	118 (51)	
Immunocompromised			<0.001
No	1186 (71)	487 (29)	
Yes	32 (46)	38 (54)	
History of cancer			0.511
No	1186 (70)	514 (30)	
Yes	32 (74)	11 (26)	
ARDS on admission			<0.001
No	1184 (77)	347 (23)	
Yes	34 (16)	178 (84)	
Pulmonary diseases			<0.001
No	1130 (73)	427 (27)	
Yes	88 (47)	98 (53)	
Complications			
Sepsis			<0.001
No	1157 (84)	219 (16)	
Yes	61 (17)	306 (83)	
Bacterial pneumonia			<0.001
No	854 (80)	209 (20)	
Yes	364 (54)	316 (46)	
ARDS			<0.001
No	1110 (92)	99 (8)	
Yes	108 (20)	426 (80)	
Arrhythmia			<0.001
No	1201 (75)	411 (25)	
Yes	17 (13)	114 (87)	
Acute kidney injury			<0.001
No	1194 (80)	301 (20)	
Yes	24 (10)	224 (90)	
Hepatotoxicity			<0.001
No	1214 (71)	488 (29)	
Yes	4 (10)	37 (90)	
GIT perforation			<0.001
No	1217 (71)	492 (29)	
Yes	1 (3)	33 (97)	
Multi-organ failure			<0.001
No	1216 (78)	349 (22)	
Yes	2 (1)	176 (99)	
Age in years (mean)	46	57	<0.001#
Duration of stay in days (mean)	10 days	14 days	<0.001#

Among the deceased inpatients, there were statistically significant differences between those who had comorbidities and underwent ICU admission and those who did not. Compared to deceased inpatients not admitted to ICU, those admitted to ICU had 30 times the mortality rate (p <0.001). Compared to the deceased inpatients who did not have ARDS on admission, those admitted with ARDS had three and a half times the mortality rate (p <0.001). Compared to deceased inpatients without pulmonary diseases, obesity, cardiac disease, and immunocompromised conditions, those having such pre-existing conditions had nearly twice the mortality rate; moreover, they had more than one and a half times the mortality rate if they had HTN or diabetes.

The prevalence of complications was significantly associated with the mortality rate. Compared to deceased inpatients without complications, those with the following complications had significantly higher mortality rates: ARDS (10 times), sepsis (5 times), multi-organ failure and acute kidney injury (4.5 times), gastrointestinal tract perforation and arrhythmia (approximately 3.5 times), hepatotoxicity (3 times), and bacterial pneumonia (2.3 times). Deceased inpatients were on average 11 years older than non-deceased inpatients, and they had a significantly longer hospital stay (14 vs. 10 days; p <0.001).

The results of univariate and multivariate logistic regression analysis (Table 4) suggested that using the central BU as a reference, COVID-19-related mortality was significantly higher in the northern and western BUs and significantly lower in the southern and eastern BUs. On controlling for other variables, the adjusted odds ratios (AORs) of the most important predictors associated with COVID-19 mortality during admission, using the central BU as a reference, were 9.90 and 1.55 times higher in the northern (95% CI 4.53-21.60, p < 0.001) and western (95% CI 1.04-2.13, p< 0.001) BUs, respectively, and 0.60 and 0.23 times lower in the southern (95% CI 0.36-0.99, p = 0.079) and eastern (95% CI 0.14-0.38, p = 0.002) BUs, respectively.

**Table 4:** Multiple logistic regression analysis of the factors related to COVID-19 deaths CI=confidence interval; AOR=adjusted odds ratio; COR=crude odds ratios; REF=reference category.

Characteristics	Multi-variate				Univariate			
	AOR	95% CI		p. value	COR	95% CI		p-value
		Lower	Upper			Lower	Upper	
Business Unit								
Central	1	REF			1	REF		
Western	1.55	1.04	2.13	<0.001	3.18	2.35	4.30	<0.001
Eastern	0.23	0.14	0.038	0.002	0.26	0.17	0.39	<0.001
Northern	9.90	4.53	21.61	<0.001	3.39	2.09	5.52	<0.001
Southern	0.60	0.36	0.99	0.079	0.51	0.31	0.83	0.006
Age groups								
18–39 years	1	REF						
40–59 years	2.96	1.87	4.68	<0.001	-	-	-	-
60 years and older	6.47	3.76	11.13	<0.001	-	-	-	-
Gender								
Male	1	REF						
Female	0.067	0.44	1.02	0.064	-	-	-	-
Nationality								
Non-Saudi	1	REF						
Saudi	0.5	0.34	0.73	<0.001	-	-	-	-
History of chronic diseases								
Diabetes	0.83	0.57	1.20	0.314	-	-	-	-
Hypertension	0.92	0.61	1.38	0.697	-	-	-	-
Obesity	1.57	1.01	2.44	0.045	-	-	-	-
Cardiac Diseases	1.40	0.85	2.32	0.188	-	-	-	-
Length of Stay	0.48	0.38	0.61	<0.001	-	-	-	-
Complications								
Bacterial Pneumonia	0.96	0.67	1.38	0.838	-	-	-	-
Multi-organ Failure	101.36	24.02	427.60	<0.001	-	-	-	-
Sepsis	27.76	17.62	43.74	<0.001	-	-	-	-

## Discussion

The new MOC's goal is to ensure that all Saudis have access to the best and most efficient quality of care while maintaining cost-effectiveness in a sustainable value-based healthcare system [30]. This study aimed to simulate and compare inpatient COVID-19-related mortality rates among the five prospective administrative BUs of the KSA to be constituted according to Vision 2030 from the consolidation of the 13 current public administrative regions [30]. The five anticipated BUs are central (our reference), western, eastern, northern, and southern. The experience of consolidating the thirteen regions of the KSA under the administration of the Ministry of Health into five BUs has shown the disparity in mortality rates as a quality indicator of healthcare in hospital admissions during the pandemic. After controlling for patient and disease factors, compared to the central BU, the northern BU had the highest mortality rate followed by the western, whose rates were 9.90 and 1.55 times higher, respectively, showing statistically significant increases, whereas the rates in the southern and eastern BUs were lowest, with rates 0.60 and 0.23 times lower, respectively, also showing statistically significant decreases. Disparities in the quality of care between BUs have been observed in ICU admissions indicating similar results with the northern and western BUs being highest and southern and eastern BUs being lowest revealing a concerning trend [17].

A possible cause of this disparity is the present structure of healthcare in the KSA. All healthcare facilities are under the administration of the MOH, making them too large and cumbersome to manage efficiently. Saudi Arabia has already undertaken an initiative to restructure the healthcare system with a new Model of Care that will make each BU accountable for equitable access to a high quality of care in all regions within the unit. This simulation will also be instructive as a model for global attempts to restructure healthcare systems to be more responsive and resilient when the next crisis hits. The purpose of healthcare systems is to treat patients with the best standard of care on time. Crises such as pandemics highlight the weaknesses in healthcare systems and, if viewed as instructional, can reform our systems to be several magnitudes better.

Our study's 30% mortality rate is higher than that from a study conducted at King Saud University Center Hospital, showing a mortality rate of 17.5% but shares the findings that variations in healthcare quality were observed in the KSA [33]. Three factors may explain differences in the outcomes of our studies. Firstly, our study participants were limited to inpatients with moderate-to-severe COVID-19 symptoms in 30 hospitals, which met the MOH criteria for hospital admission. The King Saud University Medical City-King Khalid University Hospital (KSUMC-KKUH) study included all COVID-19-confirmed inpatients in a single hospital. Secondly, we speculate that there may have been unnecessary hospitalizations of patients with milder symptoms that might have impacted or strained resource availability, burdening the healthcare system and causing medical staff to focus on non-severe cases, thus risking severe cases. Thirdly, medical cities and teaching hospitals are generally known to have the latest medical advances and resources compared to public hospitals from a quality perspective. This further indicates that implementing a new MOC that enhances competition among BUs through privatization could improve the quality and efficiency of healthcare services.

Consistent with other studies, our data showed a significantly higher risk of death in COVID-19 inpatients with pre-existing obesity and the COVID-19 complications of sepsis and multiorgan failure [34, 35]. Among our variables, we also documented that non-Saudi’s had a 50% higher mortality rate than their Saudi counterparts despite the government declaration of free treatment for all residents, including legal and illegal non-citizens [36]. Detailed study of this variable is outside the scope of this study. However, we speculate that the difference in mortality rates between Saudi and non-Saudi participants may include several factors such as lower educational levels, lower socioeconomic status, language barriers, and legal status. These factors should be studied in-depth to find the primary causes of this phenomenon which is similar to a California study finding that societal factors may account for the disparity in COVID-19 hospitalization and mortality rates among African Americans despite the state’s efforts to provide care for all COVID-19 patients [37].

Regarding the trajectory of the COVID-19 pandemic, global COVID-19 mortality rates were clearly affected by the timing and severity of infection rates and changed from pandemic phase to phase, for example, the experience in Italy and the UK where there were tremendous changes in mortality rate due to changes in preventive measures and treatment protocols [38, 39]. Fortunately, the KSA was not exposed to the same crisis situation as the Saudi government began imposing restrictions a month prior to the appearance of the first case in the Kingdom and strict restrictions remained throughout the period of our study which spans the first four months at the beginning of the pandemic in the KSA [39, 40]. These mitigation measures resulted in lower mortality rates in the KSA by January 2021 (1.7%) compared to 2.2% globally and 15% in the UK [1-5].

This is the first study to investigate COVID-19-related mortality as a measure of the quality of healthcare among all five BUs in the KSA, however, international studies have revealed regional disparities in mortality rates for other coronaviruses [10, 11]. The variation in the quality of care of inpatients with confirmed COVID-19 evidenced among the BUs in the KSA parallels international studies documenting regional variation in healthcare services in general prior to the pandemic [41-44]. The use of mortality rates as a quality indicator may be preferred to utilization rates as the latter has proven to be an insufficient measure of quality [42]. Our study adjusted for factors such as demographics, comorbidities, and COVID-19-related complications; however, other factors that are yet unclear may explain the disparities in mortality risk between BUs. Nevertheless, our findings reflect those of international studies on mortality rates that have been linked to the quality of healthcare for both general hospitals and long-term care facilities [22-27, 45-48].

The KSA has introduced Vision 2030, which intends to pursue a new MOC for the entire country. With demographic changes and increasing non-communicable diseases, the demand for more advanced healthcare services continues to rise throughout the KSA, and current projections foresee 44% of the population being over 40 and 14% over 60 by 2035 [29]. The existing public system is not able to meet this demand and is unsustainable. Partial privatization, according to the new MOC, is anticipated to improve response to demand by transferring the administration of healthcare aspects from the Ministry of Health to a holding company directing private insurance companies assigned to each of the five proposed BUs. The purpose of this new MOC is to assure equitable access throughout the KSA, emphasize primary care, and improve the quality of healthcare. Financial administration will remain within the jurisdiction of the MOH through a newly formed National Insurance Center, rendering a private/public hybrid system. Our study has shown extensive variation in the quality of care (as reflected by mortality rates), the reduction of which is an express goal of Vision 2030, which, when fully implemented, is anticipated to improve healthcare quality and efficiency [30]. Findings from this study will provide a basis for policy considerations to address variations in healthcare quality among the five BUs.

Strengths and limitations

The interpretation of our findings needs to be tempered due to certain limitations. As COVID-19 spread throughout the KSA in the first few months, different regions had peak morbidity rates at varying times. Therefore, a snapshot of inpatient cases for a four-and-a-half-month period might not have captured an accurate picture of each BU. Factors other than those included in our studies, such as infrastructure or staffing, might have been associated with mortality rate. Finally, our research's cross-sectional nature limited the relationship between the variables; hence, could not deduce causality. To offset these limitations, the inclusion of inpatient characteristics regarding demographics, comorbidities, and COVID-19 complications rendered a high degree of validity to our study by adjusting for important health factors that could account for differences in mortality between the BUs. Therefore, the findings of our study potentially serve as a baseline for healthcare quality differences that will need to be considered when assessing BU performance under the new MOC of Vision 2030.

## Conclusions

Healthcare systems have been designed to treat patients who come into the system one at a time, even during times like flu season. However, the beginning of the SARS-CoV-2 pandemic resulted in large numbers of infected people requiring hospitalization simultaneously, overwhelming the capacity of both human and technical resources leading to excessive mortality. Although the pandemic challenged the healthcare system in the KSA, it did not become so overwhelmed that it collapsed like many others globally. Nevertheless, mortality rate variations across the five BUs appear to be related to healthcare quality disparities. The mean COVID-19 related mortality rate for all participants during our study period was 30%, with extremes ranging from a low of 8% to a high of 53%. Even after controlling for important patient and disease covariates, these factors could not explain this disparity in COVID-19 mortality rates, providing additional evidence that healthcare quality variation was the primary explanation for the differences in healthcare outcomes. Further investigation is required to identify the specific structural; resource, including both human and non-human; as well as distributional factors associated with regional differences in healthcare quality. Additional research should include longitudinal studies through December 2020 to verify the variations between BUs. This study provides the impetus to initiate the needed changes to realize the full potential of the new Model of Care under privatization.
